# Induction of Potent and Long-Lived Antibody and Cellular Immune Responses in the Genitorectal Mucosa Could be the Critical Determinant of HIV Vaccine Efficacy

**DOI:** 10.3389/fimmu.2014.00202

**Published:** 2014-05-08

**Authors:** Nadia Chanzu, Beatrice Ondondo

**Affiliations:** ^1^Institute of Tropical and Infectious Diseases, College of Health Sciences, University of Nairobi, Nairobi, Kenya; ^2^The Jenner Institute, Nuffield Department of Medicine, University of Oxford, Oxford, UK

**Keywords:** HIV-1, HIV vaccines, elite controllers, long-term non-progressors, highly exposed persistently seronegative, mucosal immunity

## Abstract

The field of HIV prevention has indeed progressed in leaps and bounds, but with major limitations of the current prevention and treatment options, the world remains desperate for an HIV vaccine. Sadly, this continues to be elusive, because more than 30 years since its discovery there is no licensed HIV vaccine. Research aiming to define immunological biomarkers to accurately predict vaccine efficacy have focused mainly on systemic immune responses, and as such, studies defining correlates of protection in the genitorectal mucosa, the primary target site for HIV entry and seeding are sparse. Clearly, difficulties in sampling and analysis of mucosal specimens, as well as their limited size have been a major deterrent in characterizing the type (mucosal antibodies, cytokines, chemokines, or CTL), threshold (magnitude, depth, and breadth) and viral inhibitory capacity of HIV-1-specific immune responses in the genitorectal mucosa, where they are needed to immediately block HIV acquisition and arrest subsequent virus dissemination. Nevertheless, a few studies document the existence of HIV-specific immune responses in the genitorectal mucosa of HIV-infected aviremic and viremic controllers, as well as in highly exposed persistently seronegative (HEPS) individuals with natural resistance to HIV-1. Some of these responses strongly correlate with protection from HIV acquisition and/or disease progression, thus providing significant clues of the ideal components of an efficacious HIV vaccine. In this study, we provide an overview of the key features of protective immune responses found in HEPS, elite and viremic controllers, and discuss how these can be achieved through mucosal immunization. Inevitably, HIV vaccine development research will have to consider strategies that elicit potent antibody and cellular immune responses within the genitorectal mucosa or induction of systemic immune cells with an inherent potential to home and persist at mucosal sites of HIV entry.

## Introduction

The past three decades have witnessed the transformation of HIV/AIDS from a fatality to a chronic manageable disease but the situation remains far from ideal as the epidemic continues to spread. According to the UNAIDS report 2013, an estimated 35 million people are living with HIV, 2.3 million new infections, and 1.6 million AIDS-related deaths were documented at the end of 2012. The field of HIV prevention and treatment has progressed significantly over the years and has had a huge impact on the HIV pandemic. Anti-retroviral therapy (ART), more especially highly active ART (HAART) has been quite instrumental in the fight against HIV/AIDS and represents the most significant achievement that has transformed an epidemic that threatened to wipe out humanity (1). HAART has not only increased the lifespan of infected people (2), but has had other significant health benefits including reduced risk of opportunistic infections such as tuberculosis and AIDS-defining cancers, namely Kaposi sarcoma and cervical cancer (1, 3). Moving a notch higher, pre-exposure prophylaxis (PrEP) and post-exposure (PEP) trials demonstrate outstanding success as evidenced by significant reductions in the risk of HIV-1 infection via heterosexual and homosexual transmission, as well as injecting drug usage (4–8). In breakthrough infections, however, early treatment not only provides an immune advantage such as functional cure demonstrated in a few studies including the VISCONTI study, the Mississippi baby, and the recently reported Long Beach baby (9–12), but also dramatically reduces the risk of secondary transmission as demonstrated in studies with sero-discordant couples, where the infected partner initiates early treatment (13) and studies the prevention of mother-to-child transmission (MTCT) (14, 15).

The recent introduction of universal testing and treatment (UTT) followed by immediate initiation of ART to all those testing HIV positive irrespective of clinical stage or CD4 count (16–19) will have a huge impact on the epidemic, although the reality is that wider scale implementation will inevitably be logistically and financially overwhelming (20). Microbicides (8) and male circumcision (21–23) have also contributed significantly to the reduced risk of infection and/or transmission, and have great potential if deployed on a larger scale. Despite the tremendous progress in the field, challenges remain and a vaccine is still of top most priority. An HIV vaccine would not only ease the cost of burden of therapy globally, but will also have a huge public health impact. Experts agree that a comprehensive package combining an effective, safe, well-tolerated, accessible, and affordable HIV vaccine with HAART, and the current HIV preventive technologies would ultimately bring the epidemic to an end. Thus, a combination of prophylactic and therapeutic vaccines that prevent infection and afford superior control of viremia in breakthrough infections will significantly reduce the spread of infection and disease (3). The successful HIV vaccine candidates are expected to induce potent broadly neutralizing antibodies (bNAbs) and high titers of non-neutralizing antibodies, in addition to robust cellular immune responses with virus inhibitory effector functions in order to offer long-lasting sterilizing immunity, or in the case of breakthrough infections, to at least increase the threshold of HIV titers required for infection, reduce virus load setting point, and reduce secondary transmission rates (24–28). Most importantly, since a significantly large proportion of HIV infections are transmitted sexually, both heterosexual (29, 30) and homosexual, especially in men who have sex with men (31), prophylactic vaccine candidates should be effective at all possible portals of HIV-1 entry, with key focus on the genital and rectal mucosa (32).

## HIV-1 Vaccine Candidates: Progress so Far

Several factors that have rendered HIV vaccine design an unprecedented challenge have been extensively discussed in several independent reviews. These factors include the enormous HIV virion diversity (up to 35% in gp120) culminating in many HIV-1 subtypes/clades and circulating recombinant forms (33, 34); the well-documented immune evasion strategies (35) and immune escape (36); persistence of the virus in latent reservoirs that cannot be effectively cleared with HAART (37, 38); the immunoregulatory facet of HIV comprising memory CD4+ T-cell depletion from mucosal sites including the gut-associated lymphoid tissue (GALT) (39, 40) and MHC class 1 down-regulation by Nef (41) among others; and the overall lack of definitive correlates of immune protection coupled with imperfect correlations or discrepancies between systemic and mucosal immune responses (42–45). Despite these challenges, the field of HIV vaccine trials has evolved over the years, and currently, more than 200 vaccine trials (IAVI Clinical Trials Database 2014: http://iavireport.org/Trials-Database) have been conducted since the launch of the first ever HIV vaccine trial in the mid 1980s (46). Of these, a few vaccine candidates that showed modest immunogenicity in the initial stages of evaluation were implemented at Phase IIb and III to test their efficacy in HIV control (summarized in Table 1). These vaccines were designed to induce T cells alone (HVTN502 and HVTN503), T cells in combination with antibodies (HVTN505 and RV144), or antibodies alone (VAX003 and VAX004).

**Table 1:** **Phase IIb and III HIV vaccine efficacy trials**.

Vaccine trial	Vaccine candidate and immunogens	Specimens collected		Most significant immune response elicited	Reference
		Systemic	Mucosal	
HVTN 502/Merck 023 STEP Study (Phase IIb/prophylactic)	MRKAd5 HIV-1 *gag/pol/nef*	Serum, plasma, PBMCs	Not collected	T cell response	Buchbinder et al. (47)
HVTN 503 Phambili Study (Phase IIb/prophylactic)	MRKAd5 HIV-1 *gag/pol/nef*	Serum, plasma, PBMCs	Not collected	T cell response	Gray et al. (48)
HVTN 505 (Phase IIb/prophylactic)	VRC-HIVDNA016-00-VP/VRC-HIVADV014-00-VP	Serum, plasma, PBMCs	Not collected	T cell and antibody responses (gp140 binding IgG)	Hammer et al. (49)
RV144 (Phase III/prophylactic)	ALVAC-HIV vCP1521/AIDSVAX-gp120 B/E	Serum, plasma, PBMCs	Collected but inadequate	T cell and antibody responses (non-neutralizing antibodies to the V1/V2 loop)	Rerks-Ngarm et al. (50)
VAX 003 (Phase III/prophylactic)	AIDSVAX B/E (gp120)	Serum, plasma, PBMCs	Not collected	Antibody response (binding and neutralizing antibodies to gp120)	Pitisuttithum et al. (51)
VAX 004 (Phase III/prophylactic)	AIDSVAX B/B (gp120)	Serum, plasma, PBMCs	Not collected	Antibody response (binding and neutralizing antibodies to gp120)	Flynn et al. (52), Gilbert et al. (53)

*PBMCs, peripheral blood mononuclear cells*.

The major goal of T cell vaccines is to induce and maintain a high level of potent and fully functional effector T cells that will rapidly become home to mucosal sites, the first portal of entry, and abort early HIV infection (54). Evaluation of MRKAd5 vaccine (*gag/pol/nef*) in the STEP (HVTN502) and Phambili (HVTN503) Phase IIb trials demonstrated induction of relatively strong and durable T cell responses (47, 55); however, in both studies, the vaccine failed to prevent infection or control early viremia in breakthrough infections. Further analysis of breakthrough infections uncovered the existence of vaccine-induced selective pressure (56), suggesting a strong vaccine-induced immune response, and also highlighting the possibility that limited breadth of vaccine-stimulated responses might have impacted on the potential to contain virus replication during acute infection. A carefully designed follow-on Phase IIb clinical trial (HVTN505) that tested a multi-clade immunogen expressing *gag/pol/nef/env* was also halted for futility (49). This vaccine induced both T cell and antibody responses (strong IgG binding antibodies to gp140), as well as some neutralizing activity, but clearly these did not correlate with protection, and were instead skewed toward increased risk of HIV acquisition. Although the failure of these vaccines to protect against infection and the unexpected association with increased risk of HIV acquisition are a huge setback in the development of T cell vaccines, there is still cause for optimism, as follow-up analysis of the HIV-infected STEP study participants revealed a correlation of vaccine-induced Gag-specific T cells with reduced plasma viremia, independent of HLA influence (57). Furthermore, we have recently demonstrated induction of broad and very high magnitude, polyfunctional CD8+ and CD4+ T cell responses in a Phase I clinical trial of a T cell vaccine candidate (HIVconsv), expressing *gag/pol/vif/env* sequences that were assembled from the most conserved regions of HIV-1 (58, 59). Of key importance is the observation that HIVconsv vaccine-induced CD8+ effector T cells could recognize HIV-infected autologous CD4+ T cells and achieved up to 5.79 log_10_ inhibition of virus replication, suggesting that such vaccine-induced cytotoxic T cells may have great potential to impact post-infection virus replication. Indeed, these findings were corroborated in a challenge study where rhesus macaques immunized with SIVconsv (an equivalent of HIVconsv) showed robust and polyfunctional T cell responses that protected them from the pathogenic SIVmac251 (60). Independently, a T cell-based vaccine expressing SIV Gag was shown to elicit high magnitude, broad, and polyfunctional cellular immune responses that were associated with reduced SIVmac251 virus load set point, as well as decreased AIDS mortality (61). However, the efficacy of HIVconsv in preventing HIV-1 acquisition or lowering virus set points remains to be tested in efficacy trials, and if achieved, will be a significant milestone for T cell vaccines.

As it is speculated that sterilizing immunity against HIV-1 will largely depend on induction of potent bNAbs (in combination with strong antiviral T cell responses), antibody-based vaccines remain attractive in HIV vaccine development strategies although their potential benefit in terms of preventing HIV acquisition or controlling replication in humans is yet to be sufficiently demonstrated (51–53). These Phase III clinical trials (VAX003 and VAX004) tested the monovalent subtype B and bivalent subtype B/E rgp120 vaccines and showed induction of complex and robust immune responses comprising binding and neutralizing antibody responses to gp120 (Table 1), but no reduction in the incidence of HIV-1 was observed among the vaccinees. Although the high-risk nature of VAX003 and VAX004 trial participants might have had an influence on vaccine efficacy, the failure of these trials still highlighted legitimate limitations of antibody-based vaccines, in terms of preventing HIV acquisition or post-infection virus replication. Nonetheless, studies in non-human primates (NHPs) have provided solid evidence that bNAbs can be very effective in the control and elimination of experimental SIV or SHIV infections (62–64). This has paved way for the identification and isolation of a number of potent and broadly neutralizing monoclonal antibodies (65–71), as discussed in later sections.

Although the focus is largely on bNAbs, non-neutralizing antibodies may potentially play a significant role in HIV-1 acquisition and progression by acting via Fc-receptor-mediated binding of infected cells to trigger recruitment of effector cells with cellular cytotoxic activities such as antibody-dependent cell-mediated cytotoxicity (ADCC) or secretion of antiviral cytokines that inhibit virus replication, i.e. antibody-dependent cellular virus inhibition (ADCVI) (72, 73). Vaccine challenge studies in NHPs revealed a correlation of such antibody-dependent cytotoxicity and viral inhibition with reduced viral loads (74–77), thus demonstrating their potential involvement in protection. Furthermore, high levels of ADCC-mediating antibodies correlate with HIV suppression in elite controllers (78). The protective effect of non-neutralizing antibodies was indeed confirmed in the Thai Phase III RV144 trial, where potent non-neutralizing antibodies to the V1/V2 loop correlated significantly with protection from HIV acquisition (50, 79, 80). Additionally, correlation of ADCC with reduced risk of infection was also confirmed (79). The RV144 study tested the safety and efficacy of a prime-boost regimen comprising an ALVAC-HIV (a canary pox vector expressing HIV-1 *env/gag/pro*) prime and AIDSVAX-gp120 B/E (recombinant gp120) boost in heterosexual individuals at various levels of risk of HIV infection. These vaccines were designed to induce both antibody and cell-mediated immune responses and to complement each other in order to maximize protection. Hence, with 74 HIV infections among the placebo recipients compared to only 51 in the vaccinees, RV144 achieved 31.2% efficacy and although there was no effect on viral load following infection, it so far remains the most encouraging vaccine study to date. Thus, although highly elusive, the unsatisfactory outcomes of three large clinical trials (47–49, 55, 81) mean that now more than ever, the world is more desperate for a safe and an effective vaccine to prevent HIV infection and/or control progression to AIDS.

## Novel Vaccine Design and Delivery Strategies to Improve Immunogenicity and Efficacy

As much as poor immunogenicity might be blamed for the apparent lack of efficacy of several HIV vaccine candidates tested to date, the observation that vaccine-induced immune responses consistently waned over time suggests that even with the most immunogenic of vaccines, protection may be limited to only those individuals who get exposed to HIV-1 within the first few months following immunization. Although this can be effectively overcome by multiple booster immunizations, the costs and compliance issues will certainly be prohibitive. Therefore, novel strategies that circumvent these pre-existing hurdles are urgently required in order to design vaccines with improved immunogenicity and long-term efficacy. For instance, it is anticipated that strategies targeting the B cell maturation pathway to induce preferential maturation of naïve B cell precursors of potent and bNAbs could achieve long-term protection against HIV acquisition and dissemination (82, 83). Additionally, adjuvants that activate enzymes regulating somatic mutation, such as activation-induced cytidine deaminase (AID), could be utilized to potentially boost the chances of producing bNAbs (84). Durability of antibody responses may be enhanced by use of vectored immunoprophylaxis, an approach where immunoglobulin genes are inserted in a viral vector, which then provides life-long expression of high titers of the respective monoclonal bNAbs following a single intramuscular injection (85). The success of this strategy has been very recently demonstrated in a study using adeno-associated virus (AAV) encoding bNAbs against HIV, which was shown to induce prolonged antibody expression and long-lasting protection of humanized mice from high-dose intravenous and vaginal challenges with diverse HIV strains (85, 86).

Cytokines that can directly enhance B cell maturation into long-lived antibody-secreting cells (ASC), such as IL-4, IL-5, and IL-6, may be used as genetic adjuvants to increase NAb titers. The efficacy of this approach is documented by a Friend virus challenge study where co-delivery of adenovirus vectors encoding IL-5, IL-6, or IL-23 together with adenoviral vector expressing the Friend virus surface envelope protein gp70 (Ad.pIXgp70) significantly controlled virus replication and enhanced protection (87). In particular, mice co-immunized with IL-5 and IL-23-encoding adenoviruses produced higher titers of NAbs (87). Genetic adjuvants encoding type 1 interferons (IFNs) (88) and certain chemokines such as CCL3, CCL19, and CCL28 (89, 90) were also found to improve adenovirus vector-mediated immunity to Friend virus. Chemokines that attract specialized antigen-presenting cells can thus enhance vaccine uptake and increase the magnitude of the immune response. When adenovirus vector encoding CCL3 (a DC chemo-attractant) was co-delivered with adenovirus vectors expressing *gag/env* antigens of Friend virus, improved protection that correlated with enhanced virus-specific CD4+ T cells and higher NAb titers was observed (89). Similarly, CCL3 co-delivery of HIV antigens (*gag/pol/env*) induced higher titers of HIV-specific binding and neutralizing antibodies compared to delivery of antigens alone (89). Co-delivery of CCL19 and CCL28 significantly augmented mucosal and systemic antibody responses and also enhanced their neutralizing activity against homologous and heterologous HIV-1 strains (90). Thus, the adjuvant effect of these cytokines and chemokines could be synchronized with antigen delivery to enhance HIV vaccine efficacy.

Following the recent demonstration that contrary to the non-replicating adenovirus vector, a replicating cytomegalovirus-vectored vaccine offered superior protection of rhesus macaques from repeated mucosal challenges with the highly pathogenic SIVmac239 (91), the focus is shifting toward live vectors that induce stable effector memory rather than central memory CD8+ T cell responses (92). Seemingly, persistent but low-level replication of vaccine delivery vectors not only correlates with stimulation of potent effector memory T cells with enhanced antiviral capacity, but also provides stable immune-surveillance capable of clearing latent viral reserves (93). Such like vectors hold great promise for successful HIV vaccines, although they will need to be carefully selected to avoid induction of active disease in vaccine recipients, or possible antagonism of vaccine-elicited immune responses.

Immunological pressure exerted on HIV by vaccine-stimulated CD8+ T cells is thought to have caused early viral escape and contributed to the lack of protection among the STEP study vaccinees. Thus, vaccine approaches utilizing immunogens that are derived from conserved regions of HIV-1 (58, 59) or conserved consensus mosaics (94, 95) may possibly limit escape and offer better protection. Mosaic vaccines are designed to maximize coverage of global antigen epitopes and to therefore overcome HIV diversity by eliciting broad multi-clade immune responses (95, 96). Such increased diversity of immunogens (multiple epitopes and their variants) greatly increases the chances of matching vaccine-induced immune responses to the antigenic phenotype of the infecting founder virus or circulating HIV strains. Mosaic vaccines have shown great promise in NHP studies where comparatively superior breadth and depth of T cell responses was demonstrated (94, 97, 98). Furthermore, polyvalent mosaics were shown to induce both neutralizing antibodies and cellular responses (99), which effectively protected animals from high-dose challenge infections and were also effective at controlling breakthrough virus replication. Thus, to overcome the problem of enormous HIV diversity, while at the same time improving longevity of vaccine-stimulated immunity, a combination of replicating vectors (with an excellent safety profile) with both T cell- and antibody-inducing mosaic vaccines might be a much better strategy.

HIV infection activates immune regulatory pathways by increasing the frequency of regulatory T cells (Tregs) and generation of functionally impaired, exhausted CD8+ and CD4+ T cells which are inadequate to control virus replication and prevent disease progression (100). Thus, strategies that could enhance immunogenicity and overall vaccine efficacy, with particular emphasis on the control of breakthrough infections, may include concurrent depletion of Tregs and blockade of inhibitory pathways such as the programed death-1/ligand (PD-1/PD-L1) and the T cell immunoglobulin and mucin protein 3 (Tim-3) pathways during immunization. These strategies function to overcome negative regulation and restore immune function in exhausted T cells, and may be particularly attractive in therapeutic vaccines for HIV-1 where they could reduce virus loads and help to maintain an aviremic state (101, 102).

## Correlates of Protection Against HIV/AIDS

Despite intensive research, immunological biomarkers that could accurately predict reduced HIV-1 acquisition and transmission are not yet defined, thus making it a huge challenge to gage the potential efficacy of HIV vaccine candidates prior to the costly large-scale efficacy trials. This is further complicated by the fact that there is not a single documented case of immune-mediated HIV-1 clearance in infected individuals. Although the recent RV144 trial has provided significant clues of possible correlates of risk versus protection (79), the limited number of infected vaccinees studied makes it hard to draw definitive conclusions. Thus, attempts to define immune correlates of protection are still based on data largely arising from studies of long-term non-progressors (LTNPs) and HIV controllers, a rare group of individuals comprising both elite and viremic controllers with spontaneous HIV-1 control in the absence of treatment (103) and also from individuals who are highly exposed to the virus but remain persistently uninfected highly exposed persistently seronegative (HEPS) such as commercial sex workers, the uninfected partners of sero-discordant couples, or exposed healthcare providers. These studies have unraveled a number of protective factors which can be broadly divided into two categories, namely host/viral-related or immune-related factors, and are discussed in detail below.

### Host genetic factors that correlate with protection

Data from large numbers of non-progressor/slow progressor cohorts indicate that protective HLA alleles such as HLA-B27, HLA-B51, HLA-B57 and HLA-B58 which are associated with better virus control are overrepresented in these groups, thus implicating a very strong role for host genetics in the course of HIV infection and disease (104–106). Incidentally, genome-wide association and HLA class 1 analysis of HIV-specific T cell responses in the MRKAd5 vaccinees revealed that high-magnitude responses were associated with HLA-B27, -B51 and -B57, while HLA-B08 and -B45 were associated with lower level responses (107). Other host genetic factors such as the CCR5Δ32 and CCR2-64I mutations in chemokine receptor genes (108–110) as well as the killer-cell immunoglobulin-like (KIR) receptor polymorphisms (111–115) have also been associated with slow progression or resistance to infection (116). In fact individuals who are homozygous for the 32 base pair deletion in CCR5 are completely protected from HIV infection, and the protective function of this mutation has been recently demonstrated in the only patient to achieve sterilizing cure of HIV-1 following stem cell transplant from a CCR5Δ32 homozygous donor (117–120). Additionally, there are intrinsic host-resistant factors such as the restriction factors TRIM-5α, APOBEC3G, SAMHD1, and tetherin (121–123), which control HIV replication through various mechanisms, and any genetic alterations in their expression levels or patterns can alter the rate of HIV acquisition and progression (124, 125). However, it is imperative to note that the presence of these protective factors, chemokine receptor mutations, or HLA haplotypes *per se*, is not sufficient to confer a slow progression phenotype, as several studies indicate rapid progression of some infected individuals bearing these protective HLA and KIR genotypes. Additionally, it is known that some individuals without these protective genetic characteristics control HIV replication or persistently evade infection, thus strongly implicating alternative explanations for the attenuated disease course, such as immune-mediated protection.

### Immune-mediated correlates of protection

Studies of HIV-infected controllers indicate that robust, broadly directed, highly proliferative, polyfunctional cytotoxic CD8+ and CD4+ T cell responses target conserved regions of HIV-1 such as Gag correlate with reduced virus replication (126–134). In particular, CD8+ T cells from HIV-1 controllers display enhanced capacity to inhibit HIV-1 replication (129, 135–138) either via direct killing of infected cells or by secretion of antiviral factors known to suppress HIV replication (139). These cells also produce higher levels of IL-2 (140), have superior capacity for clonal expansion, and contain more granzyme B and perforin (138, 141). In some cases, these cells can exhibit the exceptional capacity to supress viral replication *in vitro* without prior antigen re-stimulation (127, 129). However, it is worth noting that although control of HIV-1 in various independent (but small) slow progressor cohorts correlates significantly with enhanced magnitude and breadth of T cell responses, a study looking at a larger sample size (*n* = 124) of well-defined elite controllers showed that elite control of HIV-1 was in fact associated with the lowest magnitude and breadth of IFN-γ responses, as well as the lowest titers of broadly cross-reactive neutralizing antibodies (142). This confirms that the quality of the response rather than the quantity remains important in virus control. Thus, in agreement with other studies, preferential targeting of Gag and co-secretion of both IFN-γ and IL-2 were correlated with virus control among the elite controllers than the chronic progressors and viremic controllers in this study (142).

In addition to controlling virus replication, it has recently been reported that HIV-specific CD8+ CTLs are an absolute requirement for the elimination of latent viral reservoirs following reactivation (143). Since elite controllers harbor significantly lower latent viral reservoirs (144–146), this suggests a strong link between the presence of HIV-specific CD8+ T cells with potent cytotoxic activity and controlled latency. Indeed, this was confirmed in a very recent study which demonstrated the exceptional ability of primary CD8+ T cells from elite suppressors to effectively eliminate precursors of latently infected cells (147). Moreover, elite controllers are known to develop potent effector memory (*T*_EM_) rather than central memory (*T*_CM_) T cells (148) which are not only more effective at suppressing viral control but are also known to resist apoptosis, thus capable of protecting against disease progression and establishment of latent reservoirs for extended periods in the absence of HAART (103, 149–151). Besides T cells, natural killer (NK) cells, known to inhibit HIV replication through a variety of mechanisms (152), are also numerically elevated in blood of HEPS, where they are thought to protect from HIV-1 acquisition by secretion of antiviral factors (153–155). Indeed, increased production of pro-inflammatory cytokines such as TNF-α and IFN-γ by KIR3DL1/HLA-Bw4 NK cells is associated with lower viral loads and slower progression of infected individuals (113, 156, 157). Moreover, preserved NK cell function (which is lost in progressive HIV-1 infection) has been linked with asymptomatic HIV-2 infection (158). Also, increased levels of the beta-chemokines RANTES, MIP-1α, and MIP-1β, all of which are known to bind the HIV-1 co-receptor, CCR5, is linked with protection from HIV acquisition in cohorts of high-risk women (159, 160). These chemokines have been directly associated with resistance to HIV-1 infection, reduced viral replication, and subsequently delayed disease progression (161–163).

The studies described in this section mainly relate to systemic immune responses. However, it is vital that immune-mediated correlates of protection in the genitorectal mucosa are characterized since they are indispensable in defining the overall efficacy of HIV vaccines. The next section briefly highlights the immune responses found in the genitorectal mucosa that have been correlated with protection.

## Immune-Mediated Correlates of Protection in the Genitorectal Mucosa

The observation that multiple exposures to high virus load is required for successful heterosexual HIV-1 transmission suggests the existence of robust mucosal innate immunity that must be actively involved in the control of HIV-1 infection and virus dissemination. Thus, immune responses at the genital mucosa especially those of the innate immune system provide the first line of defense and are critical in preventing HIV-1 infection (164) or destruction of HIV-1 target cells at the mucosa, before the development of adaptive immune responses. Plausibly, vaccine-mediated stimulation of components of the innate immune system such as dendritic cells, NK, and NKT cells as well as macrophages in the genitorectal mucosa could have a significant reduction in HIV-1 acquisition by production of antiviral cytokines such as type 1 IFNs or even by acting through ADCC to destroy infected cells. Alternatively, the type 1 IFNs together with other cytokines including IL-15 and IL-18 can serve to augment both innate and adaptive immune responses. However, this review will only discuss the adaptive humoral and cell-mediated immune responses to HIV-1 in the genitorectal mucosa as they form a significant part of the critical determinants of vaccine-induced immunity and might therefore hold the key to accelerating HIV-1 vaccine development. Several HIV-1 vaccine candidates tested for immunogenicity and efficacy up to advanced stages (Table 1) did not yield any mucosal data (165), especially the RV144 trial, the only one to show vaccine efficacy. As such, the definitive correlates of vaccine efficacy in the genital mucosa still remain unknown. This section discusses the various mucosal immune responses which have been linked with natural resistance to HIV-1 in HEPS or attenuated disease course in HIV controllers.

### Protective cellular immune responses in the genitorectal mucosa

A large body of evidence documents the existence of HIV-specific cellular immune responses in blood of HEPS (166–170), although there have been lots of skepticism concerning the occurrence of adaptive immune responses without active HIV infection or replication. It has been speculated that these responses arise from abortive (failed) infections that are effectively cleared before the virus successfully establishes its reservoirs. A study designed specifically to address the question as to whether HEPS truly make HIV-specific T cell responses recruited sero-discordant couples in Malawi (HPTN 052 cohort) and in the UK (CHAVI 002, St. Mary’s cohort) and used a very sensitive (cultured Elispot) assay able to detect very low frequency T cell responses (171). This study confirmed the existence of HIV-specific CD8+ and CD4+ T cell responses that were mapped to T cell epitopes frequently targeted in HIV-infected individuals. Interestingly, the responses were maintained across multiple visits in the absence of HIV infection, as no virus could be detected even with the highly sensitive transcription-mediated amplification assay (TMA). A recent study of sero-discordant couples in Uganda reported similar findings (172), thus confirming that exposure to HIV can prime adaptive immune responses that can be boosted by repeated exposures.

There is also strong evidence that mucosal HIV-specific CD8+ and CD4+ T cells modulate HIV-1 disease course, as they can control post-infection virus replication and persistence by directly killing infected target cells or secreting a number of antiviral cytokines. IFN-γ-producing HIV-specific CD8+ T cells found in the genital mucosa of HEPS were thought to be responsible for their natural HIV-1 resistance (173). This study also revealed an enrichment of IFN-γ-producing HIV-specific CD8+ T cells in the cervical mucosa as opposed to the systemic compartment, thus strongly suggesting a role in protection against HIV-1 acquisition. However, although multiple exposures to replication-competent HIV-1 is well-documented in these individuals, in the absence of confirmed productive HIV-1 infection, it is difficult to judge whether the existence of HIV-specific T cells is indeed cause of HIV-1 resistance or merely a marker of exposure. Nevertheless, human studies assessing immune responses in mucosal compartments of HIV-1 infected individuals revealed an inverse correlation of magnitude and poly-functionality of rectal HIV-1 Gag-specific CD8+ and CD4+ T cell responses and viral load (45, 174, 175), highly suggesting that mucosal T cells do exert anti-HIV activity in the rectal mucosa. Elite control of HIV was significantly associated with strong and polyfunctional T cells, secreting CD107a and MIP-1-β among other cytokines. Intriguingly, the mucosal immune responses in HIV-1 controllers were significantly higher and more polyfunctional than those in progressors, while the systemic immune responses remained indistinguishable (45). These observations highlight the potential discrepancies between blood and mucosal immune responses and further demonstrate the importance of generating protective immune responses within the local sites of virus entry.

### Protective humoral immune responses in the genitorectal mucosa

HIV-specific mucosal IgG and IgA antibodies, especially those with demonstrated HIV-1-neutralizing activity form the pillar that protects against HIV-1 acquisition, at least in cohorts of highly exposed seronegative individuals (176–180). These antibodies can act by inhibiting various mucosal HIV-1 entry pathways such as epithelial transcytosis (181, 182) and by binding to the HIV-1 virus or by neutralizing the virus to prevent infection of CD4+ T cells by primary HIV-1 isolates (183). Alternatively, they could also serve to aggregate HIV virions and inhibit movement through cervical mucus. Contrary to individuals with progressive HIV-1 infection, the IgA antibodies found in HEPS mainly recognize the conformationally conserved regions of the gp41 subunit of HIV-1 envelope, thus indicating potential for cross-clade neutralization (178, 184, 185). HIV-specific IgA with potential neutralizing activity (179, 186) has been detected in the cervico-vaginal secretions of women (176, 187, 188) and urethral swabs of heterosexual men (189) with natural HIV-1 resistance, indicating a possible role in blocking HIV-1 acquisition. In a recent study, HIV-1-neutralizing IgA was found in the cervico-vaginal secretions of highly exposed seronegative women in a prospective discordant couple cohort study (190), thus providing definitive evidence of antiviral activity in mucosal secretions after HIV-1 exposure. One major shortcoming is that maintenance of adequate levels of these antibodies requires continuous exposure to HIV-1 (186), suggesting perhaps a lack of effective formation of memory B cells. Despite the encouraging neutralization activity of HIV-1-specific IgA antibodies and the fact that secretory IgAs are a major component of the mucosal antibody responses, not many IgA monoclonal antibodies have been isolated. However, one study indicates that human monoclonal Fab IgAs directed at gp41, which were constructed by genetic engineering of mucosal cervical B lymphocytes from HEPS, exhibited good neutralization potential and were more potent at blocking transcytosis (183). The protective effect of such conserved and naturally induced antibodies implicates them as desirable components of mucosal HIV-1 vaccines, where they can potentially abort HIV-1 infection by targeting cell-free virus to prevent entry into mucosal tissues. This concept was demonstrated in an animal study where a gp41 subunit HIV-1 vaccine-induced vaginal IgAs capable of blocking transcytosis, as well as vaginal IgGs with neutralizing or ADCC activities (43). These antibodies provided sterilizing immunity in *Macaca mulatta* monkeys challenged with SHIV-SF162P3, in the absence of systemic neutralizing antibodies, again reinforcing the importance of generating immune responses in the genitorectal mucosa.

The studies described here convincingly demonstrate that these immuno-functional parameters are predictive of slow progression in a majority of HIV controllers or sterile protection in those individuals who resist HIV-1 infection despite multiple exposures to high doses of intact replication-competent HIV-1 viruses (171). Therefore, it is plausible that vaccine strategies that can induce such immune responses would have a significantly greater chance of either preventing infection or achieving functional cure in breakthrough infections. This was to some extent demonstrated in the STEP trial where vaccine-induced T cells correlated with virus control in a few of the infected vaccinees (57). Thus, collectively, these observations indicate that a myriad of factors encompassing host genetics, immunological parameters, and viral determinants act together to bring about the attenuated disease course (103, 191) or resistance to HIV. However, although it is conceivable that vaccines can be engineered to mimic protective immune responses, little can be done to influence the host genetic factors. Therefore, vaccine development efforts need to focus on the induction of mucosal (and systemic) immune responses which confer protection independent of host genetic factors.

## Correlates of Immune Protection in the Context of Vaccine Efficacy: Are They Reliable?

To put all these into the context of vaccine efficacy, it is worth a reminder that despite the documentation of immune responses that seemingly correlate with virus control in infected or highly exposed uninfected individuals, the ultimate proof of correlates of protection can only come from efficacy trials in humans. Can we say that so far the predicted correlates of protection have translated into vaccine efficacy? As an example, protection in the RV144 trial was not related to bNAbs or strong CD8+ CTL responses as both of these were not detected. Instead only CD4+ T cells, ADCC and neutralizing antibodies to the easy to neutralize tier-1 viruses were observed. Furthermore, the RV144 trial revealed that although high levels of plasma IgG antibodies targeted to the variable regions V1/V2 loop of the envelope gp120 were associated with protection against HIV-1 acquisition, envelope-specific IgA antibodies actually mitigated the effects of protective antibodies and were associated with increased risk of infection (79).

Incidentally, high levels of such IgA-binding antibodies to gp140 were also detected in the HVTN505 study vaccinees, where they are speculated to have influenced the risk of HIV acquisition (49). These observations immediately prompt the need to further investigate the role of IgA in HIV-1 acquisition since it has been significantly associated with mucosal immunity to HIV-1 in several studies of HEPS (188, 190, 192). Moreover, these observations indicate that protection from infection is not necessarily mediated by neutralizing antibodies or robust CTLs as inferred from studies in HEPS, elite controllers, and LTNPs, thus emphasizing the need to re-define correlates of protection, and perhaps keenly study the role of non-neutralizing antibodies in protection versus risk. It is possible that vaccines inducing high levels of IgG and lower levels of IgA, concurrently with broadly directed high-magnitude cellular immune responses at mucosal sites will more likely protect against infection and post-infection virus replication. It remains possible that the efficacy of RV144 may have been due in part to the presence of vaccine-induced antibody and cellular immunity in the genital mucosa.

More intriguing, however, is the fact that all the HIV-1 vaccine candidates tested for efficacy showed modest immune responses in preclinical and early stages of clinical evaluation but have shown no efficacy in the longer term. Take for instance, the MRKAd5 vaccine that induced robust responses in majority of vaccinees but failed to prevent HIV infection or post-infection viremia (47, 55). Although this vaccine was not expected to prevent infection, the fact that it did not attenuate disease course despite the observed immune responses begs the question as to whether the current measurements of evaluating vaccine immunogenicity during the preclinical and Phase I clinical trials (193) are robust enough to predict vaccine failure. Apart from the inevitable discrepancies between animal models and humans, this might also perhaps suggest qualitative and quantitative differences between the vaccine-stimulated responses and those required to prevent HIV-1 acquisition at the first point of encounter (genitorectal mucosa). Indeed, in addition to paucity of CD4+ T cell responses, the CD8+ T cell responses in the MRKAd5 vaccinees were found to be of low magnitude, narrowly directed, less polyfunctional, and targeted mostly Pol and Nef (55), contrary to what has been correlated with spontaneous HIV-1 control. Furthermore, as most vaccine-stimulated responses are tested by IFN-γ ELISPOT in peripheral blood samples, it could be though that potent responses are elicited in the systemic compartment, their migration to mucosal portals of entry where they are critical may be a limitation. Indeed, some studies have revealed a lack of correlation in the magnitude, quality, breadth, and functional capacity of T cell responses between blood (systemic) and mucosal samples (43–45). Thus, in addition to induction of robust systemic antibody and cell-mediated immunity, vaccine strategies that focus on generating local immune responses within the genitorectal mucosa, such as the “prime-pull” (194) approach may be more successful at reducing the replication and dissemination of transmitted founder viruses. Moreover, it remains possible that other parameters of the mucosal immune response such as proliferation, *in vitro* viral inhibition, and ADCC could be important (74, 111, 195, 196). Thus, accurate correlates of protection should include active sampling of mucosal compartments and measure several immunological and phenotypic parameters, including expression of mucosal homing receptors and ligands.

Recent studies document the antiviral potential of vaccine-stimulated T cells against various HIV-1 isolates (58) or SIV (SIVmac239 and SIVsmE660) (197, 198) using *in vitro* virus suppression assays (VIA). The general expectation is that VIA (199, 200), which demonstrate active inhibition of the replication of intact virus *in vitro* (or *ex vivo*) would correlate with *in vivo* inhibition of HIV replication (198, 201), and would therefore be a more accurate prediction of vaccine efficacy. In fact the *in vitro* antiviral inhibitory capacity of CD8+ T cells measured by VIA was shown to accurately predict CD4+ T cell loss during early HIV-1 infection (201). However, the *in vitro* VIA did not predict vaccine efficacy or *in vivo* inhibition of virus replication in a challenge SIV study in NHPs (197), suggesting that more robust markers to predict vaccine efficacy are needed. Alternatively, this discrepancy could be attributed to lower frequencies of vaccine-stimulated T cells within the genital mucosa, and it might be that VIA performed with T cells isolated from the genital mucosa will give a better correlation with *in vivo* HIV or SIV control. Thus, studies that assess mucosal B or T cell antiviral capacity, whether vaccine stimulated (human and NHP) or HIV induced, for example in LTNPs, elite controllers and HEPS will be important in informing research aiming to identify correlates of vaccine efficacy.

On a completely separate platform, increased susceptibility to HIV-1 acquisition in vaccinees in the STEP and Phambili studies might to a certain extent reflect the dark side of vaccine-mediated immune activation that may create readily available HIV-1 targets (55, 202, 203). This hypothesis was proven in a challenge study where immunized rhesus monkeys exhibiting higher frequencies of IFN-γ secreting T cells were more susceptible to SIV infection (203). This strongly suggests that induction of sub-optimal vaccine-specific T cells (without robust antiviral effector functions) could increase the risk of HIV-1 acquisition in vaccinated individuals. Other risk factors such as herpes simplex virus type 2 (HSV-2) infection may significantly alter the immune milieu in the genitorectal mucosa (204–206) and affect HIV-1 vaccine efficacy. Indeed, HSV2 infection correlated with a fivefold increased risk of HIV-1 acquisition in heterosexual men in the HVTN505/Phambili study (48) and in homosexual men in the STEP study (47). HSV2 infection subverts cellular immune responses directed to HIV-1 (205), disrupts mucosal integrity, and induces massive recruitment of HIV-1 targets (CCR5+CD4+ T cells and immature DCs expressing DC-SIGN) to the genitorectal mucosa (207). These observations highlight the additional challenge to clearly define benchmark features that distinguish protective versus detrimental vaccine-induced immune cells that accelerate HIV-1 acquisition and disease progression. This is more especially due to the seemingly unavoidable paradox, where imprinting a mucosal homing phenotype on vaccine-induced immune cells is critical to prevent HIV-1 acquisition, but also poses a significant risk of increased susceptibility.

## Broadly Neutralizing Antibodies are Critical Determinants for Sterilizing Immunity

The occurrence of systemic and mucosal HIV-specific neutralizing IgG antibodies is also documented in LTNPs (135, 208–210), where they were initially linked with slow disease progression. However, recent studies indicate that their role in controlling established infection is quite limited and that high titers and breadth are in fact a result of higher virus loads (211). Indeed, such bNAbs are frequently detected in a very small proportion of HIV-infected individuals known as elite neutralizers (212), but they do not prevent disease progression (213), presumably due to rapid virus escape. Thus, a major limitation of antibodies is the high escape rate, meaning that even with the most excellent bNAbs, protection may only be transient as mutational escape occurs within a relatively short time. Moreover, several studies indicate that bNAbs are found at much lower levels among the Elite and viremic controllers (142, 214, 215), and at comparatively higher levels in chronic progressors (142), not only suggesting a lack of influence on virus replication and disease progression (211, 213), but most importantly also reinforcing the fact that neutralization breadth increases with prolonged exposure to HIV. These observations, together with the fact that bNAbs take several years to develop, incited skepticism and questioning of the potential relevance of bNAbs in preventing HIV acquisition and disease progression. Nonetheless, the possibility that bNAbs could achieve virus neutralization and block virus entry if present within the genital mucosa before infection, either by passive administration or if induced by immunization has been a cause worth fighting for.

A large panel of bNAbs (both first and second generation) have thus been extensively characterized (71), and some such as PG9 and PG16 which are found within the conserved domain of the V1/V2 loop were shown to induce potent neutralizing activity on 70–80% of circulating HIV-1 isolates (66, 216). The CD4 binding site monoclonal antibody VRC01, in particular, displays very broad neutralizing activity (>90%) against primary isolates of envelope pseudoviruses (70). These monoclonal bNAbs have been quite successful in animal studies, for instance, vaginal administration of B12 or intravenous delivery of 2F5 and 4E10 mAbs protected macaques from intravaginal or intrarectal SHIV challenges (217, 218), raising the possibility that bNAbs can attenuate HIV-1 acquisition and disease progression. Indeed, this has been demonstrated in recent passive transfer studies in NHPs showing that if present at sufficient levels and well before virus challenge, then bNAbs can in fact abort infection to achieve sterilizing immunity (77, 218–221). The main caveats making it difficult to extrapolate the relevance of NHP studies to humans are (1) unlike natural HIV-1 infection, where individuals are exposed to diverse HIV-1 strains with differing neutralization sensitivities, NHP studies often use a homogeneous challenge virus that is usually highly susceptible to neutralization. (2) Some NHP studies have used high doses of antibodies that are unlikely to be attained by immunization. (3) Some NHP studies use high doses of the challenge viruses, which can significantly mask the vaccine-mediated protection. Nonetheless, several monoclonal bNAbs with potent neutralizing activity such as the PGT, PG, and VRC series, among others (66, 70, 216) afforded sterile protection at very low serum concentrations, even with high-dose challenges (85, 219, 220, 222, 223). Furthermore, bispecific bNAbs combining the inhibitory activity of an antibody directed to domain 2 of human CD4 (ibalizumab) with either PG9 (PG9-iMab) or PG16 (PG16-iMab) exhibited high potency and neutralized 118 viruses at very low (picomolar) concentrations (224). Hopefully, such remarkable neutralization breadth and potency can be achieved in a clinical setting, via immunization or passive administration.

These observations are very encouraging as they identify the particular antibody target epitopes as excellent templates for vaccine design, and also shed some light on the threshold level of antibodies required to achieve sterilizing immunity in humans. This also raises hopes that antibody vaccines based on such potent and bNAbs would achieve significant control of virus replication in infected individuals and possibly offer sterilizing immunity in healthy vaccinated subjects. However, no vaccine has induced bNAbs so far, and the only evidence that bNAbs could offer some protection in humans comes from earlier clinical studies where passive administration of the bNAbs, 2G12, 2F5, and 4E10 was shown to transiently delay viral rebound after HAART cessation (225, 226), but the protection was very limited. In a recent Phase I clinical trial of the modified trimeric V2-deleted envelope vaccine, potent neutralizing antibodies were induced in human volunteers, but these were of very limited breadth (227), despite enhanced neutralization breadth in animal studies (228, 229). Thus, the greatest challenge for antibody vaccines is to induce bNAbs that are potent enough to recapitulate the neutralization spectra observed in elite neutralizers and to neutralize many virus isolates including the most resistant, heterologous tier-2 and tier-3 viruses. The remarkable efficacy of monoclonal bNAbs in the vast majority of animal studies discussed here may partly be due to intravenous delivery, which ensures broad anatomic dissemination including the genitorectal mucosa. However, it is still uncertain whether bNAbs induced by parenteral immunization will traverse to the genitorectal mucosa, thus strongly arguing for immunization strategies to induce potent bNAbs localized within or in close proximity to the genitorectal mucosa.

## Experimental HIV-1 Vaccines Targeting Mucosal Sites

HIV-1 mucosal vaccinology is still in its infancy and remains a challenge despite the intense interest within the HIV/AIDS field. A mucosal vaccine that interferes with HIV-1 attachment and blocks subsequent steps including crossing of the epithelial barriers within mucosal surfaces to infect target cells, while at the same time inducing potent systemic antibody and cellular immunity would have a greater potential for enhanced efficacy. Although several HIV-1 vaccine candidates administered by intramuscular injection stimulate robust systemic immune responses in peripheral blood, whether or not these vaccine-elicited T cells or antibody-secreting plasma cells can migrate to the genitorectal mucosa is not well documented. This is a fundamental requirement for a successful HIV-1 vaccine, therefore immunization modalities that either generate immune responses *in situ*, i.e., within the genital mucosa, or strategies that drive recruitment of systemically induced vaccine-specific immune cells to the genital areas are highly desirable. The quality of mucosally induced vaccine-specific immune responses and the degree to which they can disseminate to other anatomic compartments largely depends on the route of vaccine administration (230–233), besides the properties of the immunogen. Thus, it is important that vaccine delivery routes are carefully selected or optimized in order to maximize immune control in multiple sites. Perhaps the most important delivery routes are those that demonstrate potential to stimulate both antibody and cellular responses in the genitorectal mucosa, as well as other mucosal sites and within various systemic compartments. Some of these characteristics have been traditionally attributed to mucosal immunization including intranasal, intravaginal, intrarectal, and oral or sublingual delivery routes (230, 234, 235). Possibly, delivery of a vaccine at one mucosal site may induce immunity at peripheral mucosal sites via the common mucosal immune system (236, 237), although this hypothesis is disputed by studies showing compartmentalization of the mucosal immune network (238).

Despite the near consensus for mucosal delivery of immunogens being the best way to trigger mucosal immune responses (230, 239), there are still controversies as to whether mucosal antigen delivery alone can effectively induce systemic immune responses. This is largely due to the tissue-specific imprinting of chemokine receptors and adhesion molecules on immune cells following activation within mucosal inductive sites. Perhaps, the sublingual vaccination route which has been shown to induce broadly disseminated mucosal and systemic immune responses (230) may be considered as a more suitable alternative for delivery of HIV vaccines. Intrarectal administration of a DNA/MVA vaccine encoding HIV immunogens was also shown to elicit both systemic and mucosal virus-specific immune responses that were associated with delayed progression to AIDS following SHIV89.6P challenge (240). Another possible alternative is to combine both intramuscular and intranasal delivery, a strategy that has been quite successful in enhancing vaccine-induced HIV-specific immune responses in both the systemic and vaginal compartments in NHP studies (241–243). These observations demonstrate that mucosally delivered vaccines undoubtedly elicit local immune responses that are capable of disseminating to other systemic compartments. In the following sections, we highlight some of the studies which have successfully employed mucosal vaccine delivery with or without mucosal adjuvants to elicit potent immune responses in the genitorectal mucosa.

### Mucosal immunization without adjuvantation

Active mucosal immunization has been shown to induce potent cell-mediated and antibody responses at the genital mucosa in animal studies (244–246). In particular, intranasal vaccine delivery induces robust antibody and T cell immune responses in the genital mucosa, possibly due to targeting of dendritic cells in multiple organs such as the respiratory system, the gut mucosa, and the spleen (247, 248). Intranasal delivery of a number of HIV vaccine approaches such as DNA, peptide, live bacterial, and viral vectors induced strong CD8+ T cell responses and/or antibody responses (comprising IgG and IgA and sometimes neutralizing antibodies in vaginal washes) in mice and macaques (233, 249–251). Very recently, studies in NHPs have demonstrated that intranasal and oral vaccine administration routes were consistently and significantly better than intramuscular administration, and elicited mucosal and systemic immune responses that protected rhesus macaques from disease progression following intrarectal or vaginal challenges (252–256). These mucosal immunization routes induced high-magnitude polyfunctional CD8+ and CD4+ T cells in the rectum and vagina, which correlated with the extent of viral control.

Although mucosal (intranasal or intrarectal) delivery of DNA vaccines enhances vaccine-specific mucosal responses, it has been suggested that the quality, longevity and peripheral distribution of memory T cell responses in the genital mucosa could be improved by systemic (or intravaginal) administration of live vaccines (231, 257, 258). Live recombinant vaccine delivery vectors introduced via intramuscular, intrarectal, oral or intravaginal routes in a prime-boost strategy induced robust HIV-specific T cell responses in the vaginal mucosa as well as in the spleen, possibly due to active systemic infection that stimulates potent immune responses (257). In this study, intravaginal delivery of attenuated recombinant Listeria monocytogenes expressing Gag (rLm-gag) as a prime in combination with replication-defective adenovirus serotype 5 (Ad5) expressing Gag (rAd5-gag) as a boost induced robust Gag-specific CTL responses in the vaginal mucosa and these persisted for at least 5 months. The persisting CTLs were of effector memory phenotype and possessed strong cytotoxic activity which protected against a vaccinia-Gag challenge.

Influenza virus targets the respiratory system and is thus well-adapted for stimulating mucosal immunity. Therefore, recombinant influenza virus vectors expressing foreign genes efficiently stimulate potent long-lasting antibody and T cell immune responses in mucosal and systemic compartments (259–261). Chimeric influenza virus expressing HIV-1 gp120 V3 loop peptide (IHIGPGRAFTYTT) induced robust NAb and CTL responses following mucosal immunization in mice (262). In a separate study, intranasal delivery of recombinant influenza expressing the gp41 ELDKWA epitope also stimulated persistent NAb and IgA responses in nasal, vaginal, and intestinal secretions (263–265). Moreover, H1N1 and H3N2 influenza viruses expressing SIV CD8+ T cell epitopes induced T cells with the mucosal homing (α_4_β_7_) integrin following intranasal or intratracheal vaccine delivery in pigtail macaques (266). Of particular relevance to induction of long-lived vaccine-specific immunity by repeated immunization, influenza virus vectors when combined with other vectors in mucosal (intraperitoneal and intranasal) prime-boost immunization protocols, have proven effective at priming HIV-specific mucosal immune responses that could be augmented by recombinant MVA in BALB/c mice (267). This demonstrates the potential utility of influenza virus vectors in effective prime-boost immunization regimens to generate mucosal immune responses in the genitorectal draining lymph nodes to combat HIV infection. The possible limitation of influenza virus vectors is the insert capacity which may limit the size of antigens that could be delivered.

Several studies indicate that poxvirus vectors can also induce mucosal immune responses to foreign antigens. In particular, some studies have reported induction of immune responses in the genitorectal mucosa [as well as in the Peyer’s patches (PP) and lamina propria], that controlled SHIV replication in mice and NHPs following mucosal immunization with recombinant vaccinia virus (268–270). Mucosal (intranasal and intrarectal) delivery of non-replicating rMVA vaccines in a DNA-prime and MVA-boost strategy also induced robust antibody and cellular immune responses in the systemic compartment as well as in the genitorectal mucosa, which controlled SHIV replication and disease progression (240, 271). Mucosal vaccination with other poxvirus vectors including NYVAC and ALVAC also induced antigen-specific responses in mucosal compartments (272).

A number of studies also demonstrate induction of long-lived mucosal immunity following systemic immunization with live virus vectors such as Ad5 and NYVAC, owing to acquisition of mucosal homing properties by vaccine-induced CD8+ and CD4+ T cells (272–275). In some cases, the immune responses elicited following intramuscular delivery were superior or equivalent to those elicited by mucosal immunization. Intramuscular delivery of the SIV antigens; gag/pol or gag/pol/env by Ad35-prime followed with Ad26-boost in rhesus macaques induced potent NAb and cellular immune responses in the periphery and within the colorectal mucosa (276). Both peripheral and mucosal immune responses, especially Env-specific IgG correlated with reduced risk of SIV_MAC_ acquisition during intrarectal challenges. This is indeed very encouraging and may obviate the need for the more invasive genital mucosal immunization methods (intravaginal and intrarectal), although factors such as activation status and the inflammatory state of the host could affect mucosal recruitment and retention, as well as memory reactivation. Additionally, any impairment in the migratory capacity of vaccine-induced immune cells, possibly because differential up-regulation or down-regulation of mucosal homing integrins would significantly affect the biological relevance of the vaccine in the genitorectal mucosa.

### Mucosal immunization with adjuvantation

Mucosal adjuvants such as the non-toxic B subunit of cholera toxin (CTB) or heat labile toxin B subunit (LT-B) are known to boost protective antibody and cellular immune responses following mucosal immunization, and could therefore impact significantly on HIV-1 vaccine efficacy (244, 277). These mucosal adjuvants have been very successful in a number of experimental animal studies. For instance, intrarectal immunization with a synthetic peptide vaccine incorporating the mutant form of heat labile toxin, LT(R192G), as an adjuvant induced mucosal and systemic SIV-specific CTL responses that correlated with viral clearance in challenge experiments (278). Furthermore, intranasal co-administration of HIV-1 envelope antigens in a DNA/MVA or MVA/MVA immunization together with cholera toxin (CT) significantly enhanced antibody and cellular immune responses in the mucosa as well as systemic compartments (279). Other adjuvants known to enhance mucosal immune responses include immuno-stimulatory CpG motifs and pro-inflammatory cytokines such as IL-1α, IL-12, and IL-18 (44, 244, 280, 281). CpG adjuvantation in particular was shown to significantly enhance vaccine-induced antibody and cellular immune responses following mucosal delivery and to provide protection from mucosal virus challenge (282–284). The glycolipid α-GalCer also shows promise as a mucosal adjuvant which could be used with DNA vaccines (285). The use of non-replicating virosome vectors, known for their intrinsic adjuvant properties and efficient targeting of antigen presenting cells (286) may be another useful delivery platform to enhance mucosal immune responses. Intramuscular and intranasal delivery of a gp41 subunit antigen grafted on virosomes was shown to protect monkeys against SHIV challenge following induction of vaginal IgA and IgG with potent transcytosis blockade activities as well as neutralizing and ADCC activities (43). Intriguingly, protection of vaccinated animals was mediated by the mucosal antibody activities and not the serum circulating HIV-1 antibodies or bNAbs, suggesting that mucosal responses can prevent HIV acquisition in the absence of other systemic responses including bNAbs. Feasibility of the virosome delivery method for induction of mucosal antibodies in humans has been recently demonstrated in a Phase I proof-of-principle study using HIV-1 gp41-derived peptides (287). In this study, both serum IgG and IgA, as well as vaginal and rectal IgG were induced, but neutralization activity was not detected. However, vaginal secretions were shown to inhibit HIV-1 transcytosis, demonstrating potential to reduce HIV acquisition.

Ultimately, the goal for mucosal HIV-1 vaccine delivery is to generate local antibody and mucosal T cells with antiviral activities, but also with intrinsic ability to disseminate systemically in order to combat HIV-1 infection and spread. Alternatively, the use of tissue-specific adhesion molecules or chemokine-mediated site-specific directed migration of vaccine-stimulated immune cells from mucosal immune inductive sites to peripheral mucosal and systemic effector sites (230, 288) may be useful delivery strategies for HIV vaccines.

## Mucosal Homing Markers on Immune Effector Cells

The migration of effector/memory T cells and ASC such as those that secrete IgA (IgA-ASC) to various extra-lymphoid tissues including the gut and genitorectal mucosa is facilitated by specific homing receptors on immune cells, together with their cognate ligands, which are expressed in the destination tissues. Migration to the gut for instance requires up-regulation of the chemokine receptors CCR9 and CCR10, as well as the mucosal integrin α_4_β_7_ (289–292). The α_4_β_7_ integrin, also known as lymphocyte Peyer’s patch adhesion molecule-1 (LPAM-1) is a mucosal homing receptor that binds MAdCAM-1, a mucosal vascular addressin selectively expressed on intestinal mucosal endothelium. CCL25, the ligand for CCR9, is expressed mainly by small intestine endothelial and epithelial cells (293, 294), while CCL27 and CCL28 (the ligands for CCR10) are expressed in several mucosal tissues. Thus, binding of these receptors to their respective ligands mediates selective lymphocyte homing to and retention within the intestinal lamina propria and the PP (295–298). Co-expression of CCR9 or CCR10 with α_4_β_7_ is therefore a characteristic phenotype of gut homing immune cells. This guided migration is important for tissue-targeted immune activities such as that demonstrated in murine rotavirus infection, where memory/effector CD8+ T cells expressing high levels of α_4_β_7_ (i.e., α_4_β_7_^hi^CD44^hi^) homed preferentially to intestinal tissues and were more effective at pathogen clearance compared to cells with α_4_β_7_^lo^CD44^hi^ phenotype (299). Moreover, expression of gut homing receptors on CD4+ T cells was shown to be important for mucosal immune reconstitution following HAART, as failed reconstitution was linked to defective homing (300).

While migration of vaccine-induced T and B cells to the gut is crucial to prevent establishment of HIV-1 reservoirs and CD4+ T cell destruction (301), migration to the genitorectal mucosa is critical for preventing HIV-1 acquisition. Although migration to these distinct mucosal sites may be governed by distinct signals, homing to the genitorectal mucosa also requires a B7 integrin, α_E_β_7_ (CD103), which is known to mediate lymphocyte recruitment to various mucosal tissues (including the genital mucosa) by binding to epithelial cadherin (E-cadherin) (302–305). A recent study reported isolation of a functional subset of HIV-specific CD8+CD103+IFN-γ+ T cells in samples from the cervical mucosa of HIV-infected individuals (306). CXCR3 expression is up-regulated following lymphocyte activation, and allows migration of CXCR3+ cells to inflamed sites where the cognate ligands, CXCL9 and CXCL10, are up-regulated in response to inflammatory stimuli. Thus, expression of CD103 and CXCR3 by activated B and T cells is likely to direct their migration to the genitorectal mucosa, especially if some degree of inflammation is induced (194).

Naïve lymphocytes express CD62L and CCR7, the major lymph node homing markers, which allow them to circulate through various lymphoid organs, under homeostatic conditions. Upon antigen encounter, they differentiate into activated cells expressing unique adhesion receptors that are imprinted based on the site of antigen exposure (307). For instance, systemic antigen exposure can confer multiple homing signatures, whereas oral exposure preferentially induces higher levels of gut homing receptors (289). This could in part be due to increased expression of retinoic acid receptors on dendritic cells and macrophages in gut-associated lymphoid tissues [PP, mesenteric lymph nodes (MLN) and intestinal lamina propria] which facilitate imprinting of gut homing properties on activated T and B cells by generating retinoic acid to up-regulate CCR9, CCR10, and α_4_β_7_ (308–311). Thus, targeting delivery of HIV-1 antigens for activation within the PP and MLN via mucosal immunization may lead to induction of α_E_β_7_^hi^/CD44^hi^, α_4_β_7_^hi^/CCR9+, or α_4_β_7_^hi^/CCR10+ immune cells with the ability to access multiple mucosal compartments, including the genital and rectal mucosa.

## Factors That Limit Assessment of Immune Responses in the Genitorectal Mucosa

Some of the difficulties arising from studying mucosal sites include the heterogeneity in frequencies and distribution of various immune cell phenotypes, especially in the female genital tract. For instance, the frequency of CD4+ and CD8+ T cells, B cells and NK cells, as well as other antigen presenting cells varies significantly between the lower vaginal mucosa, the ectocervix, and the transformation zone (312). Furthermore, these vary significantly between individuals, owing to factors such as the menstrual cycle and hormonal regulation of the immune system, including levels of IgG and IgA antibodies (209, 312, 313). Such inconsistencies, especially in the integrity of the protective mucus barrier (314–316) and the frequency of activated HIV target cells have great influence on HIV acquisition and control (317–319). Other obstacles relate to the invasiveness of mucosal sampling procedures to obtain cervi-covaginal lavage, swabs, or rectal biopsies and the accompanying time-consuming procedures for isolation of cells from the biopsies (320). Furthermore, the cell yields are characteristically very low and inadequate for comprehensive functional analysis studies. Despite these challenges, procedures to collect, process, and analyze mucosal samples in clinical trials are actively being developed by groups such as the HIV Vaccine Trials Network (HVTN) and the Mucosal Immunology Group (MIG). With such collaborative efforts, several mucosal samples including semen, saliva, rectal and cervical secretions, as well as rectal and foreskin biopsies can now be collected and tested. Sample collection methods (including cups, adsorbent wicks, or sponges for vaginal and rectal secretions) as well as cryopreservation techniques and ultrasensitive analytical assays that utilize minimal sample volumes and cell numbers are being developed and optimized.

## Concluding Remarks and Future Perspectives

A successful HIV-1 vaccine will have to stimulate both antibody and cell-mediated immune responses within the mucosal sites of transmission and in blood, while concurrently avoiding recruitment of activated HIV-1 target cells to the genital mucosa. Owing to the enormous hurdles relating to mucosal sampling methodologies and limited sample volumes, immune correlates of protection against HIV-1 in the genital mucosa have not been routinely tested during clinical trials that evaluate immunogenicity and vaccine efficacy (165). However, the field is progressing steadily and it is anticipated that routine assessment of mucosal immune responses induced by immunization will be incorporated in clinical trials. Given the detection of antibody and cellular immune responses that correlate with protection from HIV-1 acquisition as observed in HEPS and from disease progression as seen in aviremic and viremic controllers, together with vaccine-induced responses in the STEP and RV144 trials (and the ongoing RV152 follow-up study), there indeed are several clues of the sort of immune responses that would correlate with HIV-1 vaccine efficacy. All evidence assessed to date indicates that the most successful strategy will induce high titers of both bNAbs and non-neutralizing antibodies to block mucosal transmission of multiple HIV-1 isolates, together with a strong polyfunctional T cell immune response with high antiviral capacity to rapidly target and kill any HIV-1 infected cells at the genitorectal mucosa and prevent systemic spread or establishment of latent reservoirs, before virus diversification. Above all, to attain desirable efficacy levels, vaccine-stimulated responses will indeed have to be present within the genitorectal mucosa prior to HIV-1 exposure. And to maintain sustained HIV immune surveillance, vaccines will need to induce stable, long-lasting B and T cell memory within the genitorectal mucosa, perhaps by employing vectored immuno-prophylaxis (85) or sustained antigen release (321) strategies.

As far as vaccine delivery modalities are concerned, several proof-of-principle studies highlight the feasibility of inducing potent immune responses in the systemic and mucosal compartments by delivering vaccines through intranasal, intravaginal, intrarectal, and oral/sublingual routes in various combinations of heterologous prime-boost immunization strategies. Although quite few, such strategies have shown improved vaccine immunogenicity in human studies and the efficacy of these mucosally redirected immune responses needs to be evaluated in larger clinical trials. Possible concerns about induction of tolerance following mucosal immunization (288) will need to be addressed, although this could be overcome by initial systemic priming followed with mucosal boosting, or perhaps by use of carefully designed vaccine delivery and dosage regimens. Moreover, experimental studies in animals demonstrate robust responses following mucosal priming and mucosal boosting (241, 243), suggesting that the benefits of mucosal vaccine delivery may by far outweigh the risk of tolerance induction.

It remains possible that live viral vectors will be the most effective delivery method to induce potent mucosal immune responses following the most preferred, non-invasive parenteral delivery. However, until this is tested extensively and found comparable to or better than mucosal vaccines, the search for suitable delivery platforms to induce protective T cell and antibody responses and to provide a local immune barrier at the genital mucosa must continue. Perhaps the most practical way to ensure that sufficient numbers of protective CTLs home to the genital mucosa following parenteral vaccine delivery is the innovative “prime and pull” approach that was proposed by Shin and Iwasaki (194). The potential of this strategy was proven in mice studies where priming with a model HSV-2 vaccine elicit systemic T cell responses, followed by the guided migration of these T cells by applying CXCL9 and CXCL10 chemokines in the vaginal mucosa, thus leading to increased recruitment of CD8+ and CD4+ T cells (194). This immunization strategy generated a local memory T cell pool which was stable and persisted for a long time. However, when tested in the context of HIV vaccines, the prime-pull strategy achieved only a modest effect on local and systemic antibody responses (322). Despite this, the enormous potential of this strategy to significantly enhance the magnitude and longevity of HIV vaccine-induced T and B cells in the genital mucosa warrants further testing.

In conclusion, the data available in the field thus far point to the imminent possibility of a vaccine that can stimulate the greatly desired protective mucosal and systemic immune responses. It might be that a carefully selected combination of immunogens, adjuvants, delivery vectors, and immunization routes may possibly yield an HIV-1 vaccine that induces optimal activation of the innate immune system and elicit protective antibody and T cell responses in both the mucosal and systemic compartments. So far, mucosal immunization seems to hold promise as the ultimate modality to ensure sustained levels of potent antibody and cellular immune responses at the genital mucosa, where they are required to arrest initial breakthrough infections. Moreover, since systemic responses do not accurately represent local immunity at the genitorectal mucosa, comprehensive immuno-functional and phenotypic characterization of the mucosal anti-HIV-1 immune response that correlates with *in vivo* virus inhibition, together with the mechanisms involved, will be crucial to the design of an efficacious vaccine for HIV-1. These will include accurate quantification of threshold titers of the mucosal antibody and T cell responses that would be sufficient to prevent infection.

## Conflict of Interest Statement

The authors declare that the research was conducted in the absence of any commercial or financial relationships that could be construed as a potential conflict of interest.
